# Formation of homophily in academic performance: Students change their friends rather than performance

**DOI:** 10.1371/journal.pone.0183473

**Published:** 2017-08-30

**Authors:** Ivan Smirnov, Stefan Thurner

**Affiliations:** 1 Institute of Education, National Research University Higher School of Economics, Moscow, Russia; 2 Section for Science of Complex Systems, Medical University of Vienna, Vienna, Austria; 3 Santa Fe Institute, Santa Fe, NM, United States of America; 4 IIASA, Laxenburg, Austria; 5 Complexity Science Hub Vienna, Josefstädterstr. 39, A-1080, Vienna, Austria; University of Bristol, UNITED KINGDOM

## Abstract

Homophily, the tendency of individuals to associate with others who share similar traits, has been identified as a major driving force in the formation and evolution of social ties. In many cases, it is not clear if homophily is the result of a socialization process, where individuals change their traits according to the dominance of that trait in their local social networks, or if it results from a selection process, in which individuals reshape their social networks so that their traits match those in the new environment. Here we demonstrate the detailed temporal formation of strong homophily in academic achievements of high school and university students. We analyze a unique dataset that contains information about the detailed time evolution of a friendship network of 6,000 students across 42 months. Combining the evolving social network data with the time series of the academic performance (GPA) of individual students, we show that academic homophily is a result of selection: students prefer to gradually reorganize their social networks according to their performance levels, rather than adapting their performance to the level of their local group. We find no signs for a pull effect, where a social environment of good performers motivates bad students to improve their performance. We are able to understand the underlying dynamics of grades and networks with a simple model. The lack of a social pull effect in classical educational settings could have important implications for the understanding of the observed persistence of segregation, inequality and social immobility in societies.

## Introduction

Homophily is the tendency of humans to associate with others who share similar traits. It has been observed for a multitude of different traits, including gender [1, 2], race [1–3], academic achievements [2, 4, 5], genotypes [6], aggression [7], obesity [8], happiness [9], divorce [10], smoking [11], or sexual orientation [12]. Homophily is found for different types of relationships such as between spouses [13], friends [14] and co-workers [15], and occurs in a wide range of environments including kindergarten [16], large human gatherings [17], Wall Street [18], populations of hunter-gatherers [19], or virtual societies of online gamers [20, 21]. Homophily is considered as one of the fundamental organizational principles of human societies [22], and has a number of important social implications such as the origin of segregation [23] or the perpetuation of economic inequality and social immobility [24].

**Fig 1:**
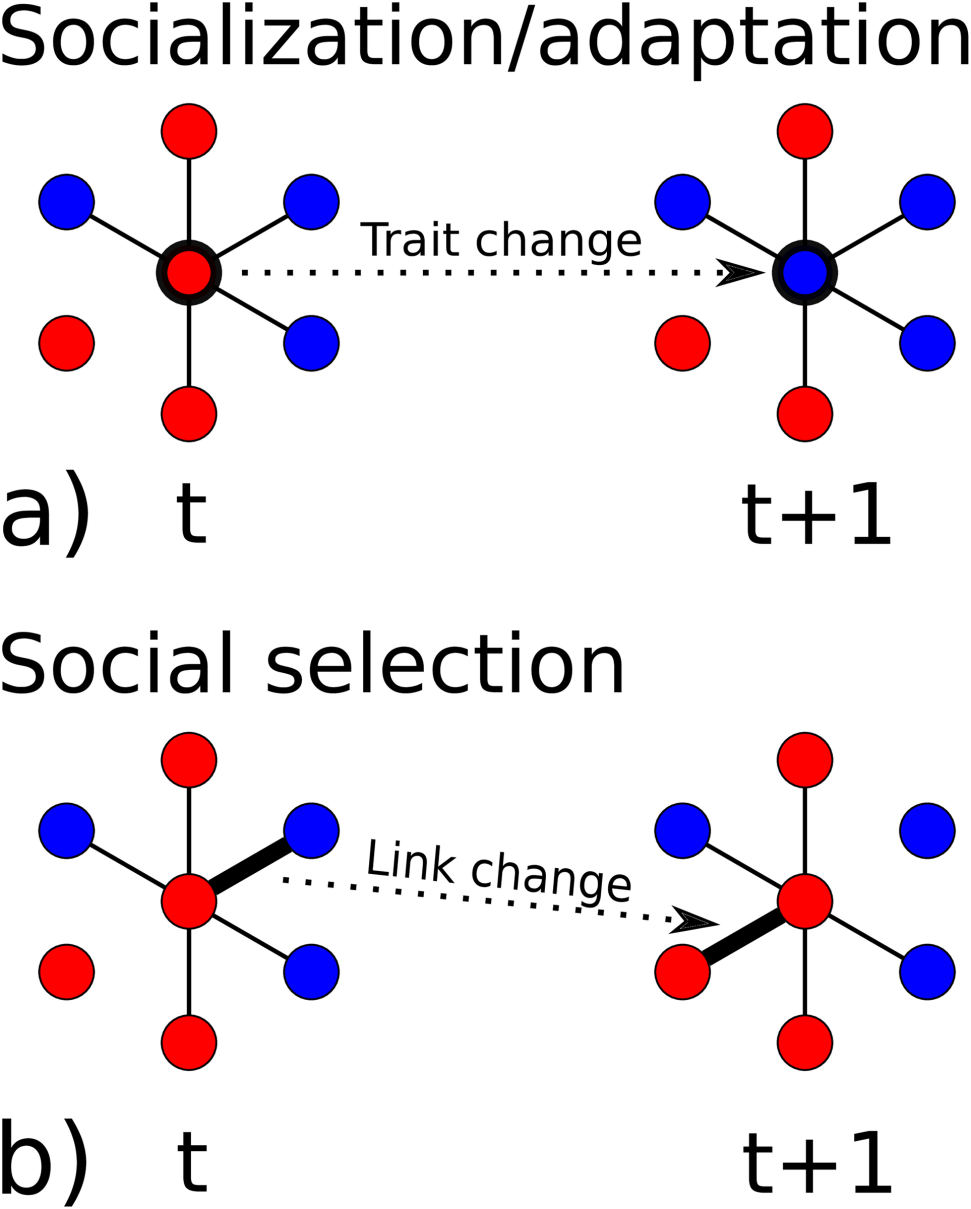
Two basic mechanisms to understand the origin of homophily. Nodes represent individuals, different colors indicate different traits. Links correspond to social ties, e.g. friendship. Increase of similarity between connected individuals may either arise from changes in traits (socialization process) (a), where individuals change their trait according to the dominance of that trait in their local social networks, or through re-wiring of their local social networks (social selection process) (b), where individuals re-shape their social contacts such that their trait matches those in the new environment better.

Even though there exists an extensive body of research on homophily, it remains a challenging question to understand its origins and how it forms and develops over time. For traits that can not be changed, such as race or gender, homophily arises through a re-structuring process of inter-human relationships, where on average links between people with similar traits are created, while links between dissimilar people are dissolved. If traits can be changed over time, the situation becomes more involved. In this case, there exist two mechanisms to explain the formation of homophily from an initially homogeneous population: *socialization* and *social selection* [25]. The mechanism of socialization means that people change their traits to increase similarity to those they are connected to in a static social environment (network). This is schematically shown in Fig 1(a). This mechanism is sometimes also referred to as ‘social contagion’ or ‘peer influence’. Under the mechanism of social selection, individuals re-arrange their social ties so that they become linked to people that are similar in traits, see Fig 1(b). If both mechanisms are at work at the same time, traits and social networks are said to *co-evolve*. The literature on the aspect of co-evolution is limited because of the lack of the simultaneous availability of longitudinal social networks and traits data.

In this paper we focus on homophily in academic achievements. It might be expected that academic performance plays an important role in friendship sorting. Homophily in achievements may arise from several factors, including school organization [26] and school policies such as ability-grouping [27] that increases the probability to meet students with similar academic performance. However, such policies are not common in the Russian education system that is characterized by its egalitarian nature and its high level of standardization. There is no tracking or ability-grouping in the particular school we use in this study. Students from that school are from the same neighborhood, the majority of them lives within 10 minutes walk from the school (see S1 Text).

Adolescence is the period of major social changes in the lives of young people [28]. Many of these changes lie in the area of peer relationships [29]. Starting from early adolescence young people become much less involved with their parents and more with their peers [30]. In this period, adolescents are concerned about their popularity among friends and seek peer acceptance [31]. To no surprise, peer influence extends to many areas of their life, including academic [32]. It was previously shown that peers may influence school engagement [33], disruptive behaviour in the classroom [34], and academic performance [35].

However, similarities between friends are not simply explained by peer influence. Adolescents also choose friends with similar behaviours and attitudes [36]. One major feature of adolescence is formation of a personal identity [37], it means that young people begin to explore and examine psychological characteristics of the self in order to discover who they really are, and how they fit in the social world in which they live [38]. Peer-group membership is also a part of identity as the group of friends to which adolescents belong helps define who they are [39]. We might expect, for example, that high performing students seek friendship with other high performing students as part of their academic identity formation. There is evidence that the importance of pears peaks in the early adolescence and then gradually declines when young people develop a mature sense of autonomy [31].

Homophily in academic performance has been studied with network data collected with questionnaire-based surveys [4, 5]. The design of these studies makes it hard to follow the temporal evolution of social networks. The availability of new technologies and big datasets provides researchers with novel tools to observe the dynamics of social networks with high temporal precision. For example, the temporal structure of social networks has been reconstructed by email data [40], computer game logs [41], or interactions on learning management system platforms [42].

The quantification of social ties remains a challenging task [43]. Even traditional approaches that are based on self-reported friendship ties may contradict the common definition of friendship. For example, it was shown that only half of self-reported friendship links was reciprocal (despite the fact that almost all of them were perceived as reciprocal) [44]. Alternatively, friendship links may be inferred from digital records of human behaviour, allowing to track the detailed evolution of social ties [45], including those among high school students [46]. We approximate friendship links between students from their activities on the social networks site, in particular from the placement of “likes” on other students’ pages. Some classical sociological theory suggests that social relationships are maintained through the symbolic exchanges [47], where exchange of “likes” may be considered as such a “symbolic ritual” [48]. Indeed, there is direct empirical evidence that “likes” correlate with real friendship ties [49].

In this paper, we use a unique anonymized dataset to observe the temporal formation of academic homophily based on social interactions between Russian students from a public high school and a university. The dataset contains information on the students’ academic performance at several time points during their studies together with the detailed information about the evolution of their friendship networks (see S1 Text). These networks between students were obtained from the largest European social network site VK (http://vk.com), that provides a functionality similar to Facebook. VK users create their profiles with information about their identity, education, interests, etc. The use of the real name is required by VK. Users may indicate other users as their friends. VK friendship is mutual and requires confirmation. However, using VK friendship links is not the most efficient way to study the dynamical evolution of actual friendships, since only information about the current friendship is available, which makes it practically impossible to extract the dynamics of VK friendship links. It is also impossible to distinguish active friendship ties from obsolete ones since VK friendship links are rarely dissolved. We, therefore, approximate friendship links between students from the placement of “likes” on other students’ pages. A link from one student to another is created if a “like” was placed at least once within a given period of time (see S1 Text). This approximation of actual friendships by social interaction strengths allows us to track the effective network evolution between students with much higher precision (see S1 Fig).

**Fig 2:**
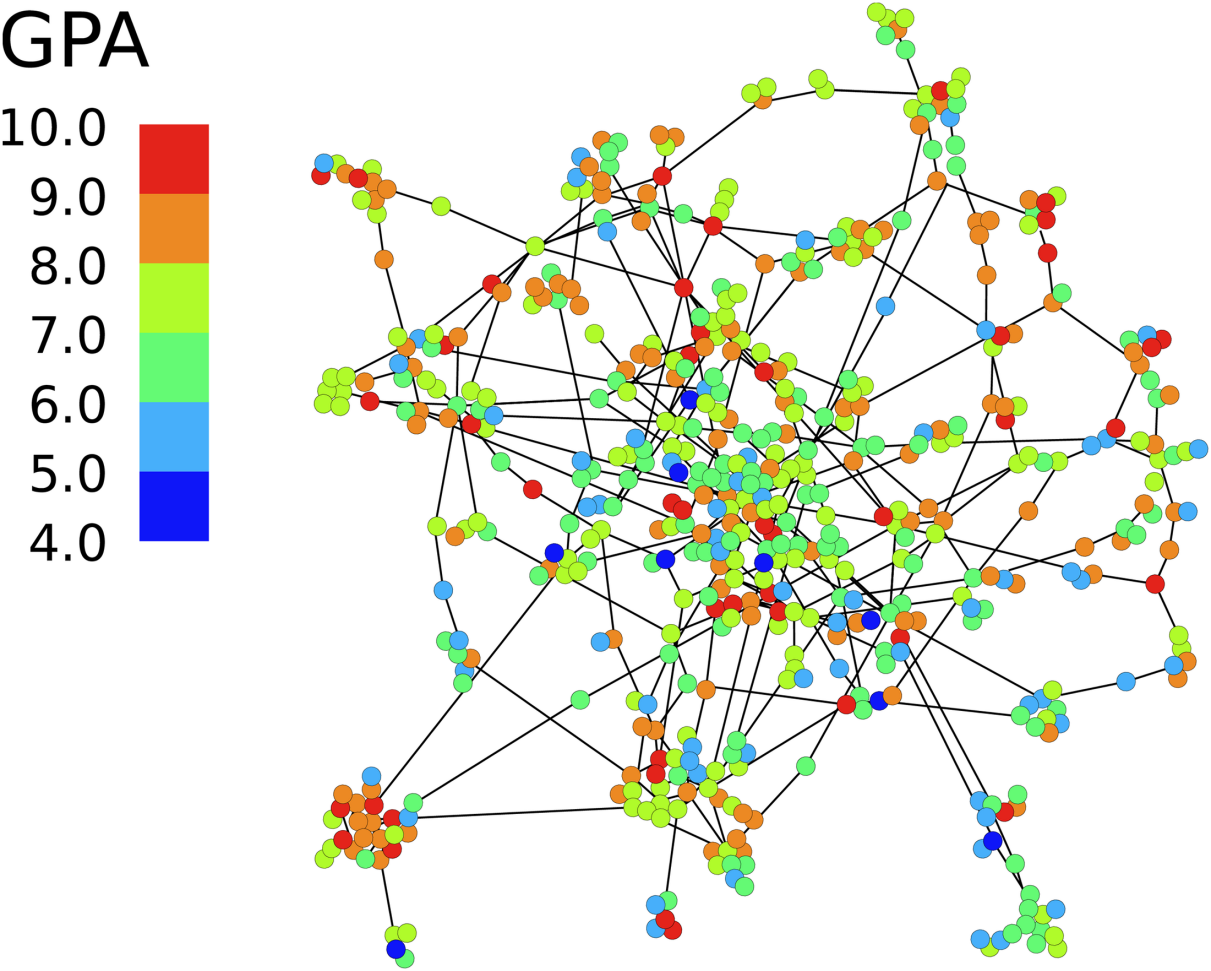
Snapshot of the friendship network of university students. The network is reconstructed from students’ interactions on the social network site VK, the Russian variant of Facebook. Nodes represent students, links exist if one student gave a “like” to another at least once in March 2016. Color represent the performance (GPA) of students across the whole period of studies. There is visible clustering of students with similar GPA.

The network of university students (seniors) on March 2016 is shown in Fig 2. Previous studies on the Facebook (or its Russian analog VK) have focused on the (relatively static) friendship marking options that are provided by the sites [50–52]. This dataset not only allows us to quantify the extent of academic homophily among students but also to see its detailed evolution over time. In particular, we are able to clarify the mechanism behind the emergence of academic homophily from an initially homogeneous population across several years.

We use two datasets of academic performance records measured as grade point averages (GPA), one with 655 students from the 5th to 11th grades (age from 11 to 18) of a Russian public high school in Moscow (for reasons of anonymity we do not state the name of the school), the other with 5,925 bachelor students of the Higher School of Economics in Moscow. High school students receive their grades at the end of each trimester, their GPAs for the last 5 trimesters were available. Since the academic year of 2014/15 the Higher School of Economics started to publish a public ranking of its students. It contains information about their GPAs for the current semester along with the aggregated average GPA across the whole period of their studies. We collect the temporal GPA data into a vector $G_{i}^{\text{HS}/U}\left(t\right)$ that represents the GPA of student *i* at time *t* and corresponds to a student’s performance within the time period from *t* − 1 to time *t*. *t* = 1 indicates the end of the first trimester/semester, *t* = *T* is the end of the last trimester/semester for a given group of students. HS indicates “high school”, U is “university”. For detailed information about time points corresponding to GPAs collection see S6 Fig. Note that grades are different for high school and university. For high school grades range from 2 (worst) to 5 (best), for university from 4 (worst) to 10 (best). The average GPA of a student across the entire available time period we denote by ${\bar{G}}_{i}^{\text{HS}/U}$. For university students we follow 4 cohorts that are labeled by *X* in the following way: $G_{i}^{U,X}$, where *X* = 1, 2, 3, 4 stands for freshmen, sophomores, juniors, and seniors, respectively. The average GPA for high school students and the cohorts of university students are presented in S1 Table.

To generate a proxy for the temporal friendship interaction network between students we use the popular SNS VK, whose main component is a user-generated news feed. This feed contains all content that was generated (posted) by users and is generally visible to friends only. If users like the content that was posted by their friends they can indicate this by an instant feedback called a “like”. “Likes” may mean different things to different people [53], however, “likes” can, in general, be seen as an indication of *active* friendship contacts between users.

VK provides an application programming interface (API) that allows to download information systematically in an open JSON format. In particular, it is possible to download user profiles from particular educational institutions and within selected age ranges. For each user, it is possible to obtain the list of their friends and the content that was published by them along with the VK identifiers of users that liked this content. Posting times are known with a time resolution of one second. “Likes” for specific content are almost always placed within 1-2 days after the content was posted. Using specially developed software the profiles of students of a given institution were downloaded and automatically matched by their first and last names with the available data on students’ performance. 88% of all high school students and 95% of university students could be identified on VK (see S1 Text). The matching procedure was performed by authorized representatives of the high school and the Higher School of Economics, respectively. After the matching procedure, all names and VK identifiers were irrevocably deleted. The “likes” of all users were collected with corresponding timestamps, those from users outside the educational institutions were removed. “Likes” were then aggregated to intervals of 3 months periods. For each group of students, we obtain a *N* × *N* adjacency matrix *A*(*t*), where *A*_*ij*_(*t*) = 1 if student *i* places at least one “like” to student *j* from time *t* − 1 to time *t*. For detailed information about time periods corresponding to collected network data see S6 Fig. The subsequent deletion of all information on individual “likes” and respective timestamps prevents the possibility of any de-anonymization. The resulting datasets were transferred to the Institute of Education, which made it available for research in fully anonymized form.

## Results

**Fig 3:**
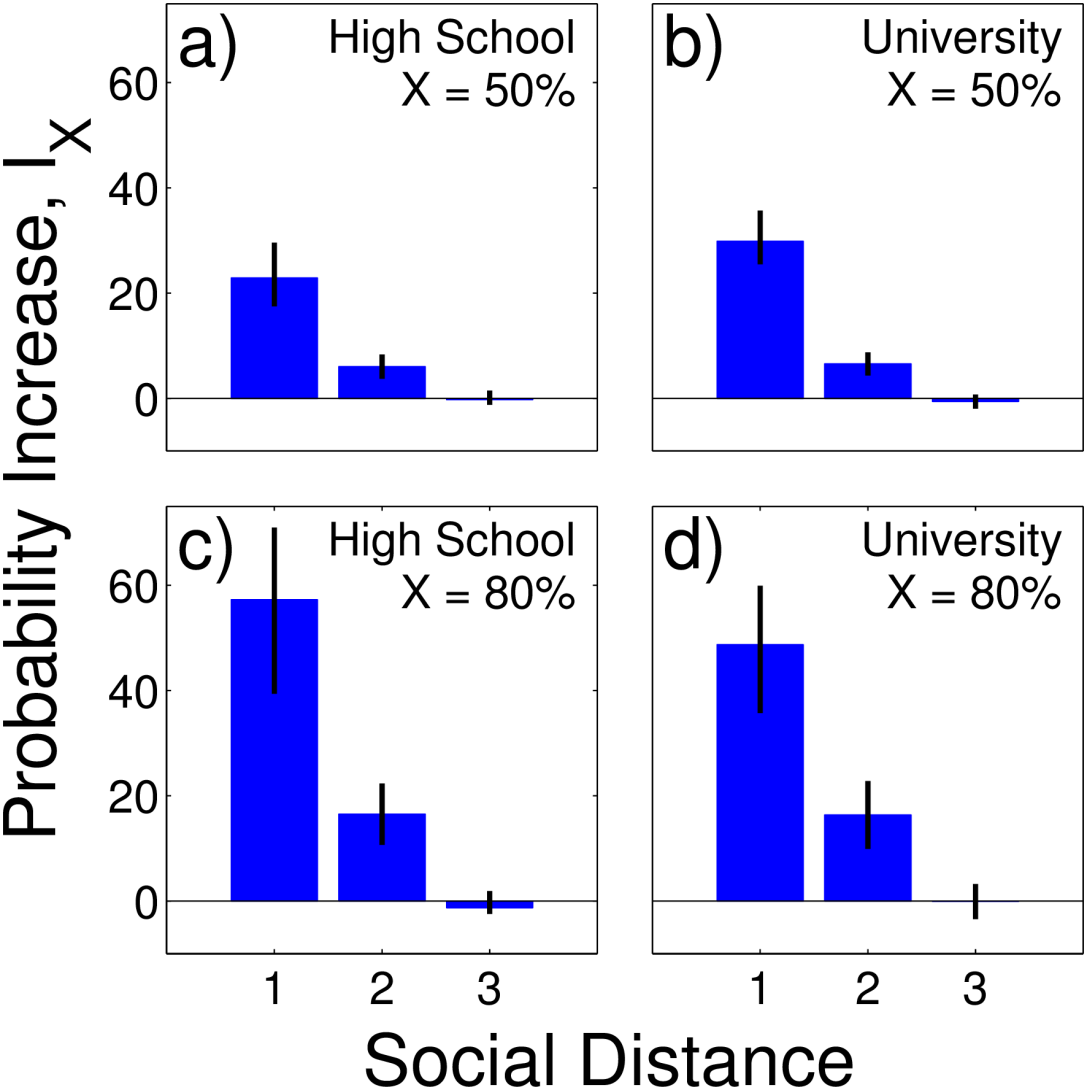
Homophily of students with good (a) and (b), and excellent grades (c) and (d), as a function of social distance. Observed increase in probability *I*_*X*_ that a student is in the top *X*th percentile of students, given that their friends are also in the top *X*th percentile. Results for the high school are shown in (a) and (c), for university in (b) and (d). Vertical lines indicate 95% confidence intervals computed with the permutation test. The social distance of 1 means friends, the social distance of 2 means friends of friends and the social distance of 3 means friends of friends of friends.

We first demonstrate the existence of academic homophily and then try to understand its origin. For all groups of students we find strong homophily. To make it comparable with other homophily studies such as in [14], we use a standard way of quantifying it by the conditional probability increase, *I*_*X*_ that a student belongs to top *X*th percentile of performers, given that his/her friends also belong to the same percentile (see S1 Text). *I*_*X*_(*t*) = 0 means that grades and friendship network are uncorrelated, *I*_*X*_(*t*) = 100% means that the probability to be in the top *X*th percentile is 2 times higher if the student’s friends are also in the top *X*th percentile, compared to the situation when they are not. *I*_*X*_(*t*) can not only be computed for friends (social distance 1) but also for friends of friends (social distance 2), and friends of friends of friends (social distance 3), etc. In Fig 3 we fix *X* to be the 50th (above average students) (a) and (b) and 80th (excellent students) percentile (c) and (d), respectively. For social distances up to 2 we observe significant homophily for all student groups at the last time point *T* = 6 for high school, and *T* = 14 for university. We find $I_{50\%}^{\text{HS}}\left(6\right)=23\%$, $I_{80\%}^{\text{HS}}\left(6\right)=57\%$, $I_{50\%}^{U,4}\left(14\right)=30\%$, $I_{80\%}^{U,4}\left(14\right)=49\%$, *p*-value < 10^−4^. Significance was tested with a permutation test (10,000 permutations), see Methods. Note that the corresponding values at the first time point are smaller, $I_{50\%}^{\text{HS}}\left(1\right)=22\%$, $I_{80\%}^{\text{HS}}\left(1\right)=34\%$, $I_{50\%}^{U,4}\left(1\right)=16\%$, $I_{80\%}^{U,4}\left(1\right)=28\%$.

This result holds independent from the method used. Following an alternative approach for scalar variables we compute the *assortativity coefficient* *r* [54] and again find highly significant homophily at the last time point *r*^HS^(6) = 0.20 (*p*-value < 10^−4^) and *r*^U,4^(14) = 0.21 (*p*-value < 10^−4^). At the first time point homophily is much smaller, *r*^HS^(1) = 0.12 and *r*^U,4^(1) = 0.12.

**Fig 4:**
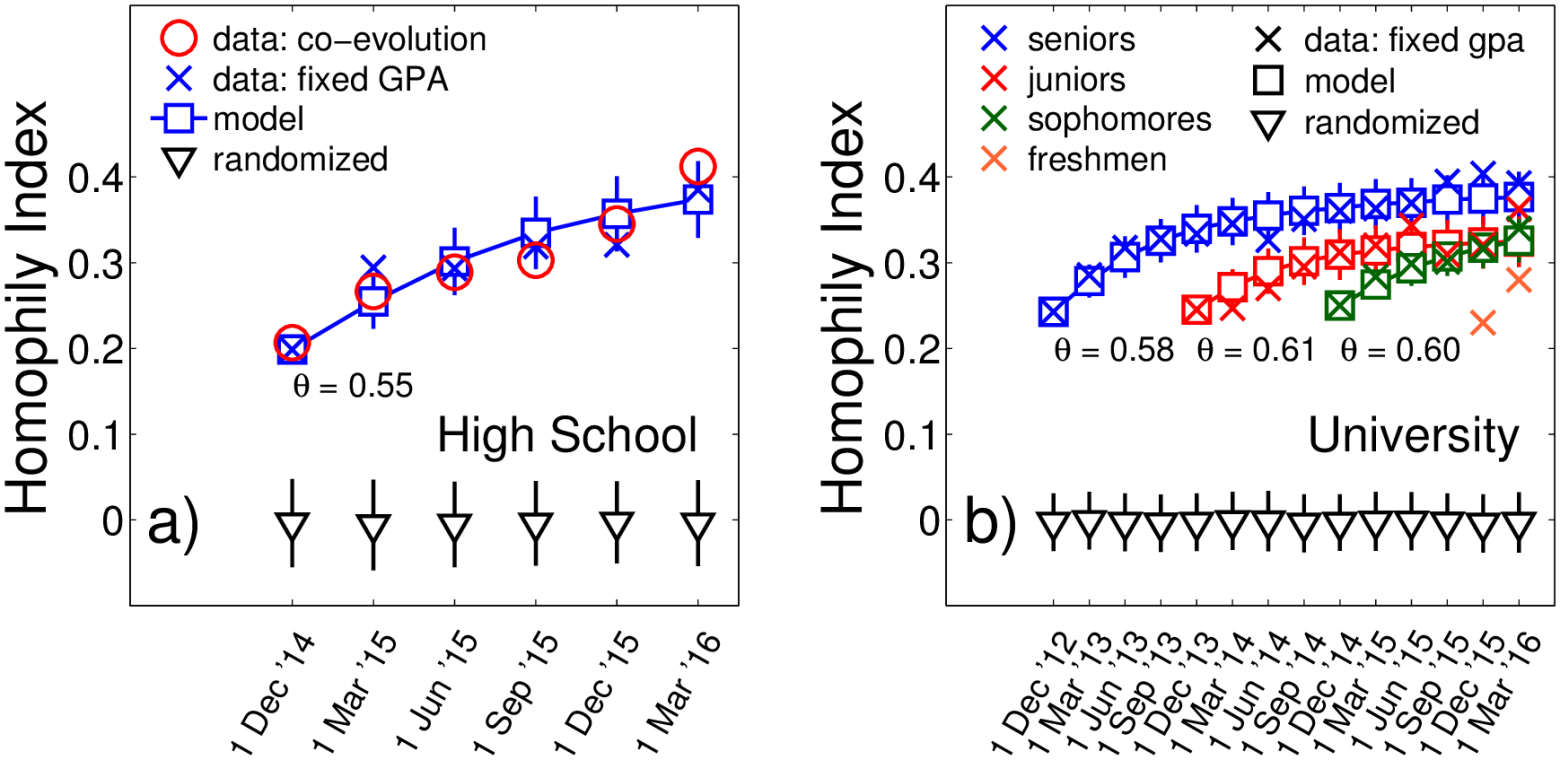
Evolution of homophily (Homophily index) in friendship networks of high school (a) and university students (b). Homophily increases with time by almost a factor of 2 (circles). The significance of the observed effect is measured with a randomization test (triangles), where grades were reshuffled randomly between the nodes in the network. It is amazing that when the GPAs of individual students are fixed to their temporal average (crosses), practically the same increase of homophily is observed, which signals the dominance of network restructuring. Results can be understood with a simple model (squares). Vertical bars are standard deviations.

In Fig 4 we show the time evolution of homophily over 1.5 years for high school students (a) and over 3.5 years for university students (b). We employ a transparent definition of a *Homophily Index*, *H*(see Methods). We see a clear increase of *H* from the first to the last trimester from about *H* = 0.20 to *H* = 0.41 for the high school (a) (circles), and from *H* = 0.24 to *H* = 0.40 for university (b) (crosses). We next show in a series of three arguments that the increase in homophily over time can not be explained by the socialization/adaptation mechanism, i.e. by the changes in GPAs over time.

### Ruling out socialization/adaptation

1. The first argument why socialization/adaptation is not the relevant mechanism behind the observed homophily increase, is due to the fact that academic performance is known to be a relatively persistent feature of students. It was shown that school-entry academic skills have large predictive power for later academic performance [55], and that academic performance might be heritable [56]. We find the persistence of performance in our data. The average GPA over high school students (3.85 ± 0.55) and its variance do practically not change over time, see S2 Fig. The average absolute difference between two consecutive time points 〈|*G*_*i*_(*t*) − *G*_*i*_(*t* − 1)|〉_*i*_ is 0.130, which means that the variation between GPAs of the same student at different time points is much smaller than the variation across students. Similar results are observed for the university students, with an average GPA of 7.41 ± 1.03 and a mean absolute difference 0.40.
2. The second argument why the socialization/adaptation mechanism can be ruled out is due to the observation that if we fix the GPAs for the high school students and do not let evolve them over time (we use the average GPA over all trimesters ${\bar{G}}_{i}$), we observe practically the same homophily increase as for the co-evolving GPAs, Fig 4(a).
3. Finally, we use a regression model to explain the GPA of students *G*_*i*_(*t*) by the explanatory variables: GPA at the previous trimester/semester *G*_*i*_(*t* − 1), by the influence of friends’ GPAs, by gender and by age (see S1 Text). The results are presented in S2 Table. For high school and university alike we find that the coefficients for *G*_*i*_(*t* − 1) (*α*_1_) and gender (*γ*) are significant and the coefficient for friends’ GPA (*α*_2_) is not. Again, this suggests that GPAs are rather stable over time and are almost fully determined by the GPA at the previous time point. The regression shows no evidence for an adaptation effect.

### Social selection and network re-organization

Due to the second argument above the explanation of the observed homophily increase can only come through changes in social networks over time, i.e. the social selection mechanism, where students preferentially select new friends that are similar in performance. A simple model allows us to understand the situation. It assumes that whenever students select new friends they prefer students who are more similar to them than their current friends. Every student *i* is endowed with a fixed GPA ${\bar{G}}_{i}$ (constant). There exists an initial friendship network that we initialize with the observed network at timestep 1, $A_{ij}^{\text{model}}\left(1\right)=A_{ij}\left(1\right)$. From time *t* to *t* + 1 the model runs through the following steps

- Pick a student *i* at random,
- Pick a random friend *j* of *i*, ($A_{ij}^{\text{model}}\left(t\right)=1$),
- Pick a random potential new friend *k* ($A_{ik}^{\text{model}}\left(t\right)=0$),
- If *k* is closer to *i* than *j*, i.e. if |*G*_*i*_ − *G*_*k*_| ≤ |*G*_*i*_ − *G*_*j*_|, rewire the link from *ij* to *ik*. Otherwise, rewire the link from *ij* to *ik* anyhow, with probability *θ*,
- Repeat until all students are updated, then continue with next timestep until *t* = *T*.

Clearly, if *θ* = 0, rewiring happens only if a potential friend is closer in GPA than a current one (strict homophily increase); if *θ* = 1 we have pure random rewiring. For a fixed *θ* we compute the Homophily Index *H*^model^(*t*) based on model networks. *θ* is fitted from the data such that $\sum_{t=1}^{T}{\left(H^{\text{model}}\left(t\right)-H\left(t\right)\right)}^{2}$ is minimized.

We find *θ* values within the range of 0.55 and 0.61 for all groups. This means that students choose new friends among those who are similar about 64%-81% more often than among those who are not similar. The results of the model are presented in Fig 4 (boxes). The experimental homophily increase is recovered. Remarkably, for all student groups, the model is able to reproduce even details in the empirical GPA distances between stable, discontinued, and new friendships, see S3 Table.

**Fig 5:**
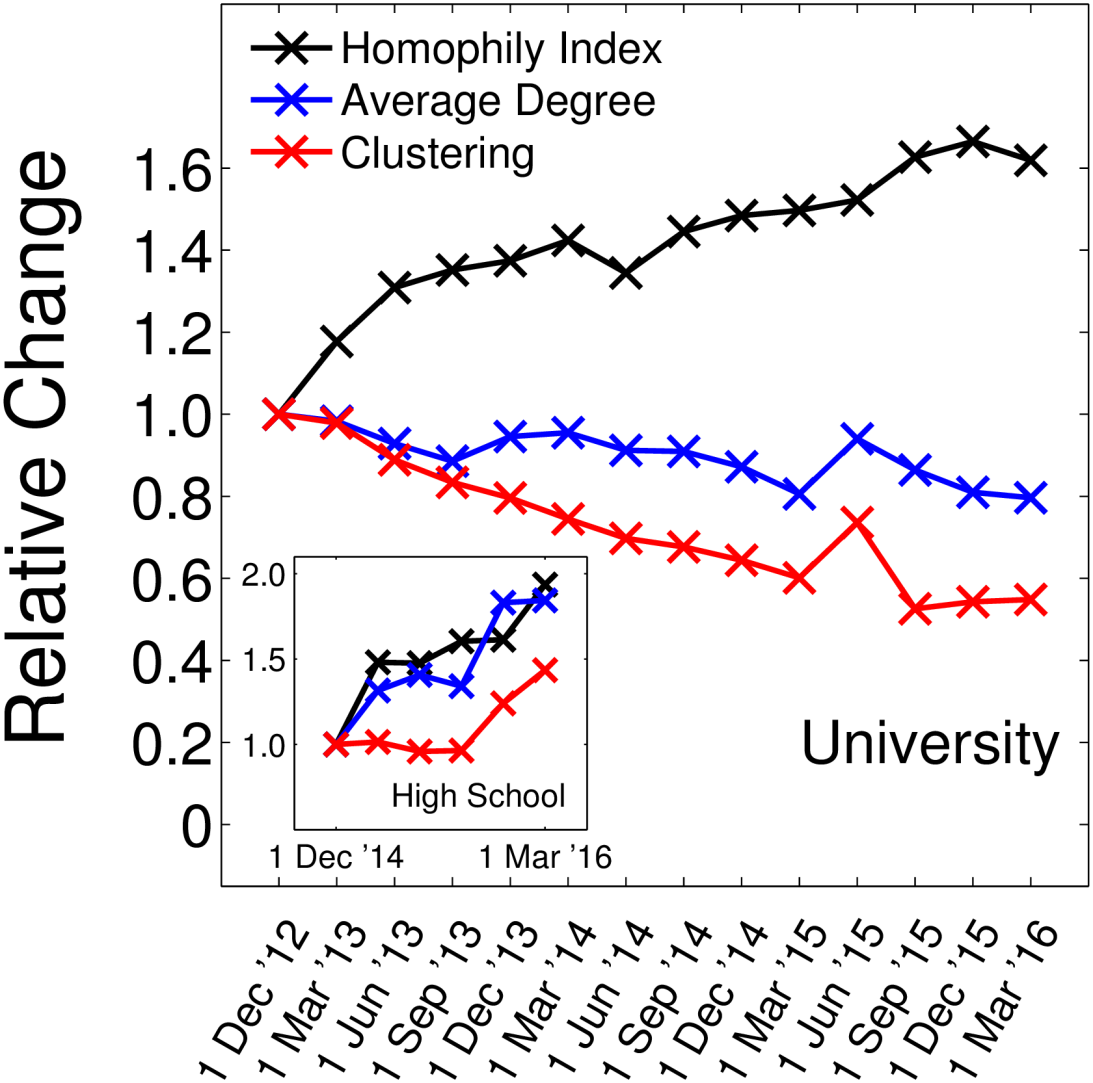
The network properties degree and clustering change over time (relative changes are shown, first time point is 1). While the network of seniors becomes sparser, there is a densification of the high school network (inset). Therefore degree and clustering coefficients can not be the drivers behind the observed homophily increase in both groups.

We have to show that the homophily increase is not explained as a trivial consequence of network densification. In both datasets we observe that friendship networks are dynamically changing over time. In Fig 5 the relative change of the average degree and the clustering coefficient of the networks are shown in comparison with the relative change of homophily. To see that the observed homophily increase is not a trivial consequence of network densification, observe that while for the high school degree and clustering increase, for university (seniors) they decrease. In both cases homophily increases. This is a first indication that degree and clustering are not the drivers of homophily change. As a second indication we test if *H* and *I*_*X*_ are significant with respect to a permutation test that preserves network topology. This is indeed the case (see Methods). Thirdly, by re-defining time intervals in a way that for each time interval the average degree is approximately the same, we find the same homophily increase (see S3 Fig), indicating that the degree is not an explanatory variable.

Finally, in S4 Fig we show that there exist slight gender differences in the homophily increase. While both genders show about the same increase over time, the homophily index *H* is slightly larger for females in the sophomore and senior groups, and larger for males for the high school students and juniors. However, the noise in our data is too large to confirm that homophily indeed peaks in early adolescence, as seen in [57].

## Discussion

We studied a unique dataset containing the academic performance of high school and university students together with detailed information about the evolution of their social ties. In accordance with previous research [2, 4, 5, 50] we found strong homophily in academic performance. The strength of academic homophily is found to be stronger than for homophily in sexual activity [58] or alcohol abuse among adolescents [59] but weaker than for homophily in smoking marijuana [59], or for age [54].

We are not only able to demonstrate the strong homophily in academic performance but also to monitor how it emerges from a homogenous population and how it solidifies over time. We show that the observed gradual homophily increase can be explained predominantly by the process of social selection, meaning that students re-arrange their local social networks to form ties and clusters of individuals that have similar performance levels. We could exclude the alternative explanations of social adaptation and co-evolution of social ties and performance. With a series of tests we ruled out the possibility that the increase of homophily results from adapting their academic performance to the one by their close friends. As an important consequence, this means that there are no indications for a pull effect, where groups of friends with good grades stimulate poor performing friends to increase their performance. The opposite effect of a negative group influence on students is also not found. It can be concluded that academic homophily in the studied groups arises and strengthens almost entirely through network re-linking.

We are able to understand the social-selection based homophily increase with a simple dynamical one-parameter model. The estimate of the parameter from the data means that students choose a new friend among those who are similar to them 64%-81% more often than dissimilar ones.

Note that even though this model is much simpler than others previously used [60], remarkably it is able to recover the increase over the whole time period for all groups, and even allows to understand details of the dynamics. It would be interesting to see in further work if these findings hold more generally also true for other student groups with different social contexts and in different countries.

It is important to note that the observed changes in social ties might be driven or facilitated by various factors. In the absence of ability tracking, other institutional factors may play a role in the segregation by academic achievements. For example extracurricular activities may provide an additional opportunity for similar individuals to meet and to from friendship ties [61]. Future research is needed to clarify the role of such specific factors.

Our findings might shed light or even confirm that access does not necessarily lead to equity. We find indications that physical mixing of students in the same educational institution does not lead to a homogeneous mixing of social ties. Even if the initial distribution is rather homogenous, students constantly re-organize their social network during the studies, which eventually results in segregation by academic performance. It is possible to conjecture that this mechanism is potentially reinforced by the accessibility of modern information technologies where maintaining links does not require physical presence anymore.

Academic achievements are the result of various factors, ranging from innate abilities to teacher qualification and family background. Regardless of these factors, it is the achievements that have direct implications for the students’ future success. For example, Russian universities select students solely on the basis of their final school examination. Thus, academic achievements determine which students are selected for elite universities and which are not. As social networks play a crucial role in social mobility [62, 63], a selective university may provide a unique opportunity to create ties that will benefit students in the future. However, if initially low-performing students from a disadvantaged background predominantly create ties with other lower-performing students it significantly reduces their upward social mobility and may explain the persistence of inequality in societies.

## Methods

### Homophily index

We introduce a Homophily Index, *H*, as the Pearson correlation coefficient between the vector of students’ GPAs, *G*_*i*_(*t*), and the vector of the average of the GPAs of their direct friends,

$$\begin{array}{c} H\left(t\right)=\text{corr}(G_{i}\left(t\right),\frac{\sum_{j}\;A_{ij}\left(t\right)G_{j}\left(t\right)}{\sum_{j}\;A_{ij}\left(t\right)}). \end{array}$$ (1)

If students’ grades are independent from average grades of their friends than *H*(*t*) = 0. Positive *H*(*t*) means that better average grades of friends lead to better average grades of students and negative *H*(*t*) means that better average grades of friends lead to worse students’ performance. *H*(*t*) = 1 means a linear relation between students’ performance and average performance of their friends.

### Randomization test

One of the challenges in understanding correlations of traits between connected individuals is to test if the observed homophily effect is significant or if it results trivially from the topology of the underlying network. To test for this we employ a typical permutation test, see e.g. [14], where we preserve the network topology and randomly reshuffle the assignment of the GPAs to the node. We repeat this procedure 10,000 times to obtain a distribution of the measures *H* and *I*_*X*_. We can then test the null hypothesis that GPAs are independent of network topology, and to compute corresponding *p*-values.

## Supporting information

S1 Text (PDF)

S1 Fig The average number of interaction (”likes“) per day between university students is presented for each cohort. The maximum observed value is 200 or 0.13 “likes” per day per student. The steep increase in September marks the beginning of studies. Some students knew each other before the matriculation.
(TIF)

S2 Fig Average GPA for high school and university students (inset) over time. Results are shown as mean values ± standard deviations. Females have better grades on average. GPAs and their variance do practically not change with time.
(TIF)

S3 Fig In the high school data the network is getting more connected over time. It is therefore possible to re-define new time intervals in such a way that for each time interval the average degree in the network is approximately the same. Clearly the homophily index *H* increases as before, indicating that the degree is not an explanatory variable. The same argument holds for the clustering coefficient.
(TIF)

S4 Fig There are no consistent differences in gender. While both genders show about the same increase over time, it is larger for females in the sophomore and senior groups, and larger for males for the high school students and juniors.
(TIF)

S5 Fig Homophily increase varies from subject to subject. Since there are only 4 possible values of grades (scores) possible for the individual subjects, we expect to observe less stable results than for the GPA. However, the general pattern of homophily increase over time holds, for mathematics it is not much pronounced.
(TIF)

S6 FigTime schedule of data collection for university (a) and high school (b) students.Network data is in the form of adjacency matrices *A*_*ij*_(*t*), where *A*_*ij*_(*t*) = 1 means that student *i* gave at least one “like” to student *j* from time *t* − 1 to time *t*. The time period from *t* − 1 to *t* is equal to 3 months. (a) For the university students (seniors, juniors, sophomores) the aggregated average GPA, ${\bar{G}}_{i}^{U}$, from the beginning of their studies on the 1st of September (2012/2013/2014) until the 1st of March, 2016 is collected. This period is equal to 3.5 years for seniors, 2.5 years for juniors and 1.5 years for sophomores respectively. The temporal GPA data, $G_{i}^{U}\left(t\right)$, was also collected for the last 3 semesters for all 3 cohorts (arrows). (b) For the high school students the temporal GPA data, $G_{i}^{HS}\left(t\right)$, is collected at the end of each trimester for the last 5 trimesters (arrows). As students do not study in summer, we assume the same performance at that period as at the last available time point i.e. spring, $G_{i}^{HS}\left(3\right)=G_{i}^{HS}\left(4\right)$. ${\bar{G}}_{i}^{HS}$ is computed as the average over the 5 trimesters.(TIF)

S1 Table Descriptive statistics of students’ GPA scores across the whole period of their studies. 〈.〉_*i*_ means average over all students in the group. Mean values and standard deviations (in brackets) are presented. Females have better grades than males on average.
(PDF)

S2 Table Coefficients from the regression model. The GPA at the current time point is almost fully explained by the GPA at the previous time point. The influence of gender is also significant, males have lower grades also after controlling for their previous GPA. The average GPA of friends at the previous time point is not significant.
(PDF)

S3 Table Re-organization of the students’ network over time. The GPA distance for new friends is consistently and significantly smaller (tested with two-sample Students’ test) than the GPA distance for discontinued friends in the observed data. Comparable results are obtained with the model.
(PDF)
