# Correction: Formation of homophily in academic performance: Students change their friends rather than performance

**DOI:** 10.1371/journal.pone.0189564

**Published:** 2017-12-07

**Authors:** The PLOS ONE Staff

## Notice of Republication

This article was republished on November 15, 2017, to correct an error in the S1 Text file that was introduced during the typesetting process. The publisher apologizes for the error. Please download this article again to view the correct version.
