# Supplementary material for: Formation of homophily in academic performance: Students change their friends rather than performance
Source: PLoS One. 2017 Aug 30;12(8):e0183473. doi: 10.1371/journal.pone.0183473 (PMC5576666; doi:10.1371/journal.pone.0183473)
Supplement: S1 Text — (PDF) [file pone.0183473.s001.pdf]

S1 Text

Details on the Education system in Russia

The Russian education system is a progeny of the Soviet system that was characterized by its egalitarian nature and high level of standardization. Children start their compulsory education at the age of 6-8 in Russia. The first 9 years of studies are mandatory. A significant number of students continue their education for two more years. Entrance tests to the first grade in schools are forbidden by law. Instead, admission to a school is based on how close a child lives to it. Each residential address is assigned to a certain school. For higher grades, there are several so-called "magnet" schools. These are high performing selective schools that attract students from all over the city. The school in our study does not belong to this type of schools. Its students live on average within 10 minutes walk from the school. Less than 5% of students need 20 minutes or longer to commute to the school.

In Russian schools, the cohort system is usually in place. After the admission students are placed randomly into several classes and stay in the same fixed group from elementary school throughout their high school years. The school schedule is fixed for the whole cohort, students do not choose which classes they take. There is generally no ability grouping or tracking.

Academic performance records of high school students

Our dataset contains academic performance records of 655 students of a Russian public school. Records include the information about all students from the 5th to 11th grades. From the 5th to 9th grades the average size of the cohort is 108, 44% of students are girls and 56% are boys. For the last two grades the average size of the cohort is smaller and the gender balance is reversed. The average cohort size is 56, 56% are girls, 44% are boys.

At the end of each trimester, students receive grades for each school subject. Grade can be 2 (not passed), 3 (passed), 4 (good) and 5 (excellent). To assess the average performance GPA is computed for 8 subjects which are present for all cohorts of students (mathematics, physics, informatics, biology, Russian, English, literature, history).

The data $G_i^{\mathrm{HS}}(t)$ was collected for the 3 trimesters of the academic year of 2014/15 and for the first 2 trimesters of the academic year of 2015/16 (see S6 Fig (b)). As students do not study in summer, we assume the same performance as at the last available time point i.e. spring. That is indicated by the equal sign in S6 Fig (b). $\bar{G}_i^{\mathrm{HS}}$ is computed as the average over the 5 trimesters. Note that when we compute average we do not count spring grades twice. The average GPA for all students and a comparison between males and females are presented in S1 Table.

Academic performance records of university students

The Higher School of Economics in Moscow ranks its students according to their performance. For that purpose, the GPAs of each student is computed. The composition of courses that are used to compute GPA varies from student to student as they are free to choose different courses. The university assigns different weights to different courses to produce the resulting GPA and we use this value "as is".

The university started to publish the ranking of its students openly on its website since the academic year 2014/15. It publishes the academic performance for the current

semester and the average GPA, aggregated from the beginning of studies to the present day. In March 2016, these cumulative GPAs for the whole period of studies $\bar{G}_i^{\mathrm{U}}$ were collected. As indicated in S6 Fig (a), this period is equal to 3.5 years for seniors, 2.5 years for juniors and 1.5 years for sophomores respectively. Our dataset also includes GPAs for individual semesters $G_i^{\mathrm{U}}(t)$, $t = 1, ..., 3$. $t = 1, 2$ corresponds to the first and second semesters of the academic year of 2014/15 and $t = 3$ corresponds to the first semester of the academic year of 2015/16 (see S6 Fig (a)). For freshmen GPAs are available at a single time point corresponding to the first (and only) semester of their studies. The grades are ranging from 4 (worst) to 10 (best). The data contains information about 1,579 freshmen (49% females), 1,570 sophomores (56% females), 1,539 juniors (52% females) and 1,237 seniors (53% females). The average GPA for each cohort is presented in S1 Table.

VK data and sampling bias

VK is the largest European social network site with more than 100 million active users. It was launched in September 2006 in Russia and provides a functionality similar to Facebook.

According to the VK Terms of Service: "Publishing any content on his / her own personal page including personal information the User understands and accepts that this information may be available to other Internet users taking into account the architecture and functionality of the Site".

There exists the following sampling bias in the data downloaded from VK. The students that were not identified on the VK are more likely to be males and tend to have lower scores. The proportion of boys among high school students that were not identified is 58%, and among identified students is 54%. The proportion of males among not identified university students is 75% and among identified students is 47%. The GPA for not identified high school students is 3.77, 3.85 for identified and for university it is 7.00 for not identified and 7.20 for identified students. The proportion of males is significantly higher for not identified university students. However, this bias should not be relevant for the results, as these populations represent less than 5% of the total population.

Homophily measures

In addition to the Homophily Index as defined in the main text, we use two standard ways to quantify homophily.

1. The conditional increase in probability is one of the standard ways to quantify homophily for binary variables and was used to demonstrate homophily in obesity, smoking, sleep, heavy drinking, alcohol abstention, marijuana, happiness, loneliness, depression, smiling in profile picture, divorce [1]. In the same spirit, from the GPA data $G_i(t)$ we can define binary variables, $G_{X,i}^{\mathrm{bin}}(t) = 1$, if student i ranks above the Xth percentile of GPA, and $G_{X,i}^{\mathrm{bin}}(t) = 0$, if the student ranks below.

   We define $P_X^+(t)$ as the conditional probability that a student is above the Xth percentile of GPA given that his or her friend is also above the Xth percentile,

$$P_X^+(t) = \frac{\sum_{\{(i,j)|G_{X,i}^{\mathrm{bin}}(t)=1, G_{X,j}^{\mathrm{bin}}(t)=1\}} A_{ij}(t)}{\sum_{\{(i,j)|G_{X,j}^{\mathrm{bin}}(t)=1\}} A_{ij}(t)} \quad . \tag{1}$$

   Similarly, $P_X^-(t)$ is the conditional probability that a student is above the Xth

percentile of grades given that his or her friend is below the Xth percentile

$$P_X^-(t) = \frac{\sum_{\{(i,j)|G^{\mathrm{bin}}_{X,i}(t)=1, G^{\mathrm{bin}}_{X,j}(t)=0\}} A_{ij}(t)}{\sum_{\{(i,j)|G^{\mathrm{bin}}_{X,j}(t)=0\}} A_{ij}(t)} \quad . \tag{2}$$

We then define the *conditional increase* in probability, $I_X(t)$ as the fraction,

$$I_X(t) = \frac{P_X^+(t) - P_X^-(t)}{P_X^-(t)} \times 100\% \tag{3}$$

2. For scalar variables one standard way to measure homophily is the Pearson correlation coefficient r across all friendship pairs which is sometimes called *assortativity coefficient* [2].

$$r = \frac{\sum_{ij}(A_{ij} - k_i k_j/M)x_i x_j}{\sum_{ij}(k_i \delta_{ij} - k_i k_j/M)x_i x_j} \quad , \tag{4}$$

where δ_{ij} is a Kroneker delta, and $M = \sum_{ij} A_{ij}$.

GPA distance

We define the average *GPA distance* between friends for a given vector of their GPA, $G_i(t)$, and a given fixed friendship network F_{ij} as

$$D_F(t) = \langle |G_i(t) - G_j(t)| \rangle_{\{(i,j)|F_{ij}=1\}} \quad , \tag{5}$$

where $\langle . \rangle_{\{i,j\}}$ means average over all pairs of i and j satisfying the condition. We then consider several groups of friends.

Discontinued friends are students that are friends at time $t = 1$, but are not friends at time $t = T$. $F_{ij}^{\mathrm{disc}} = 1$ if $A_{ij}(1) = 1$, and $A_{ij}(T) = 0$.

New friends are students that are friends at time $t = T$ but are not friends at time $t = 1$. $F_{ij}^{\mathrm{new}} = 1$ if $A_{ij}(1) = 0$ and $A_{ij}(T) = 1$.

Stable friends are students that were friends both at time $t = 1$ and $t = T$. $F_{ij}^{\mathrm{st}} = 1$ if $A_{ij}(1) = 1$ and $A_{ij}(T) = 1$.

Then we can compare GPA distances between discontinued friends $D^{\mathrm{disc}}(t) = D_{F^{\mathrm{disc}}}(t)$, new friends $D^{\mathrm{new}}(t) = D_{F^{\mathrm{new}}}(t)$, and stable friends $D^{\mathrm{st}}(t) = D_{F^{\mathrm{st}}}(t)$. In the same manner we define the distance for a fixed GPA,

$$D_F = \langle |\bar{G}_i - \bar{G}_j| \rangle_{\{(i,j)|F_{ij}=1\}} \quad . \tag{6}$$

To compare GPA distances between new D^{new} and discontinued D^{disc} friends we perform a two sample Students' t-test.

Regression model

To understand the influence of different covariates on students' performance, we use a simple regression model (7). We assume that students' GPAs at time t depends on their GPA at the previous time step $t-1$, on the average GPA of their friends at the previous time step $t-1$, on their gender and the years of studies[1],

[1]Some authors suggest to include GPA of friends for several time lags (t and $t-1$ or $t-1$ and $t-2$) to be able to disentangle network peer influence from social selection. However, it was argued that this approach alone can not disentangle the two mechanisms [3].

$$G_i(t) = \alpha_1 G_i(t-1) + \frac{\alpha_2}{k_i(t)} \sum_{j=1}^{N} A_{ij} G_j(t-1) + \sum_{\kappa} \beta_\kappa Y_{\kappa,i} + \gamma S_i + c + \epsilon_t \quad , \tag{7}$$

where $k_i(t)$ is the degree (number of friends) of student i at t, and ϵ_t denotes white noise. S_i is a binary variable representing the gender of a student (1 corresponds to male) and $Y_{\kappa,i}$ are set of binary variables, $Y_{\kappa,i} = 1$ if student i is currently at the κ year of its studies, and $Y_{\kappa,i} = 0$, otherwise.

To account for multiple observations of the same individual we use generalized estimating equations (GEE) [4]. A GEE is used to estimate the parameters of a generalized linear model with a possible unknown correlation between outcomes. To compute p-values for the parameters estimates we use a Wald test [5].

References

1. Christakis NA, Fowler JH. Social contagion theory: examining dynamic social networks and human behavior. Statistics in medicine. 2013;32(4):556–577.
2. Newman M. Networks: an introduction. Oxford university press; 2010.
3. Shalizi CR, Thomas, AC Homophily and contagion are generically confounded in observational social network studies. Sociological methods & research. 2011;40(2):211–239.
4. Liang K-Y, Zeger SL Longitudinal data analysis using generalized linear models. Biometrika. 1986;73(1):13–22.
5. Wald A Tests of statistical hypotheses concerning several parameters when the number of observations is large. Transactions of the American Mathematical society. 1943;54(3):426–482.
