# Supplementary figures and images for: Formation of homophily in academic performance: Students change their friends rather than performance
Source: PLoS One. 2017 Aug 30;12(8):e0183473. doi: 10.1371/journal.pone.0183473 (PMC5576666; doi:10.1371/journal.pone.0183473)

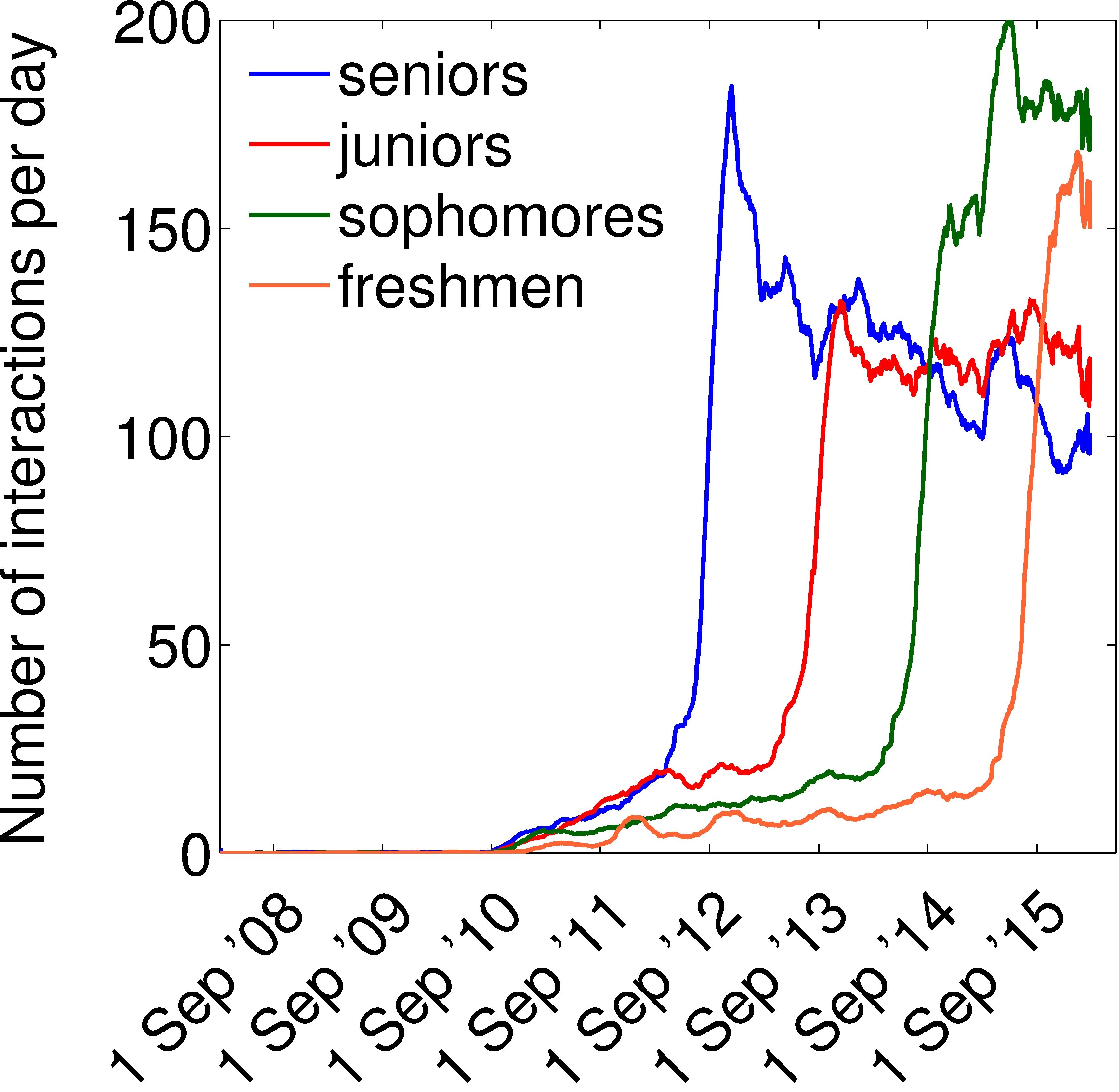

Supplement: S1 Fig — The maximum observed value is 200 or 0.13 “likes” per day per student. The steep increase in September marks the beginning of studies. Some students knew each other before the matriculation. (TIF) [file pone.0183473.s002.tif]

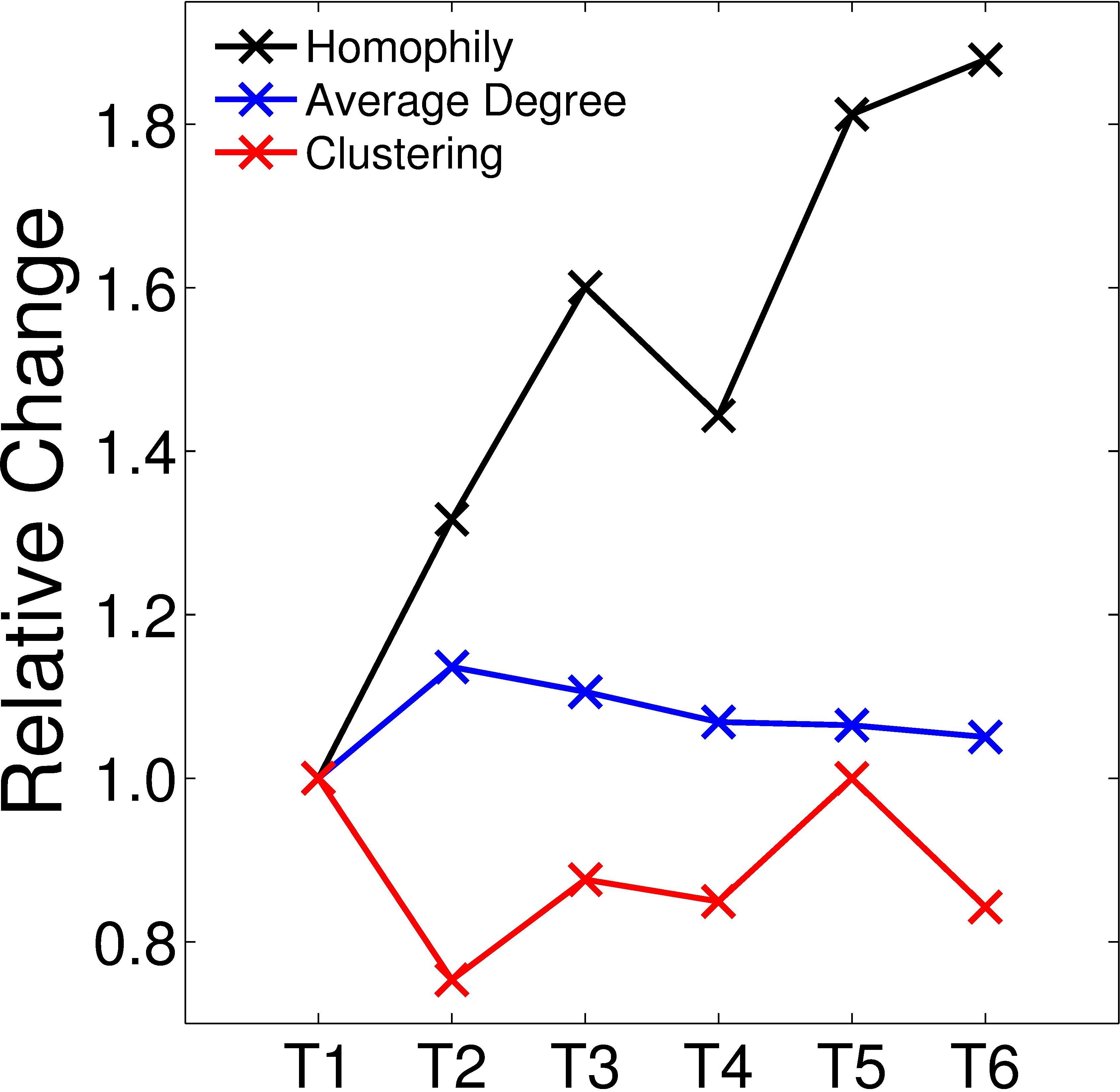

Supplement: S2 Fig — Results are shown as mean values ± standard deviations. Females have better grades on average. GPAs and their variance do practically not change with time. (TIF) [file pone.0183473.s003.tif]

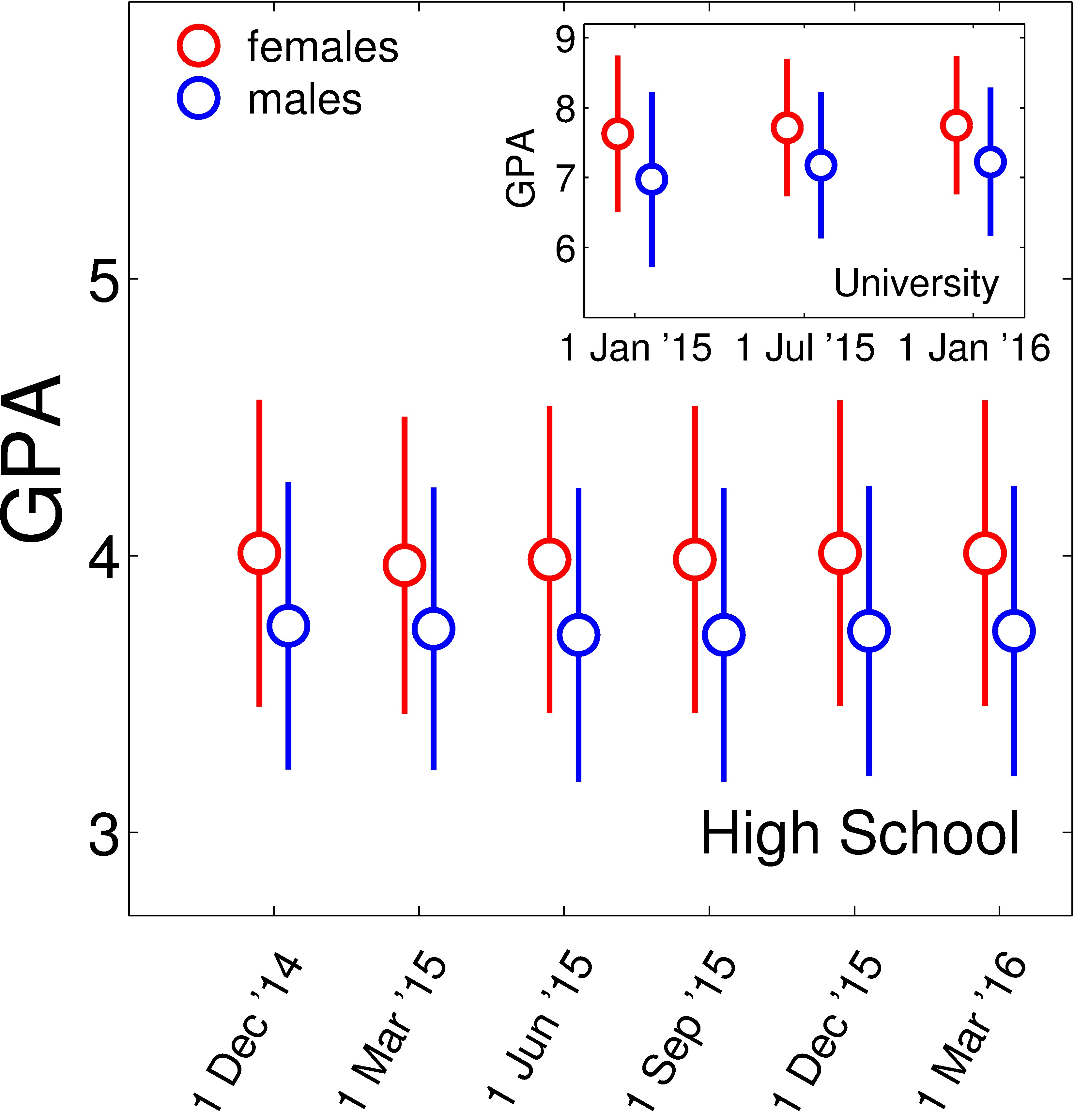

Supplement: S3 Fig — It is therefore possible to re-define new time intervals in such a way that for each time interval the average degree in the network is approximately the same. Clearly the homophily index H increases as before, indicating that the degree is not an explanatory variable. The same argument holds for the clustering coefficient. (TIF) [file pone.0183473.s004.tif]

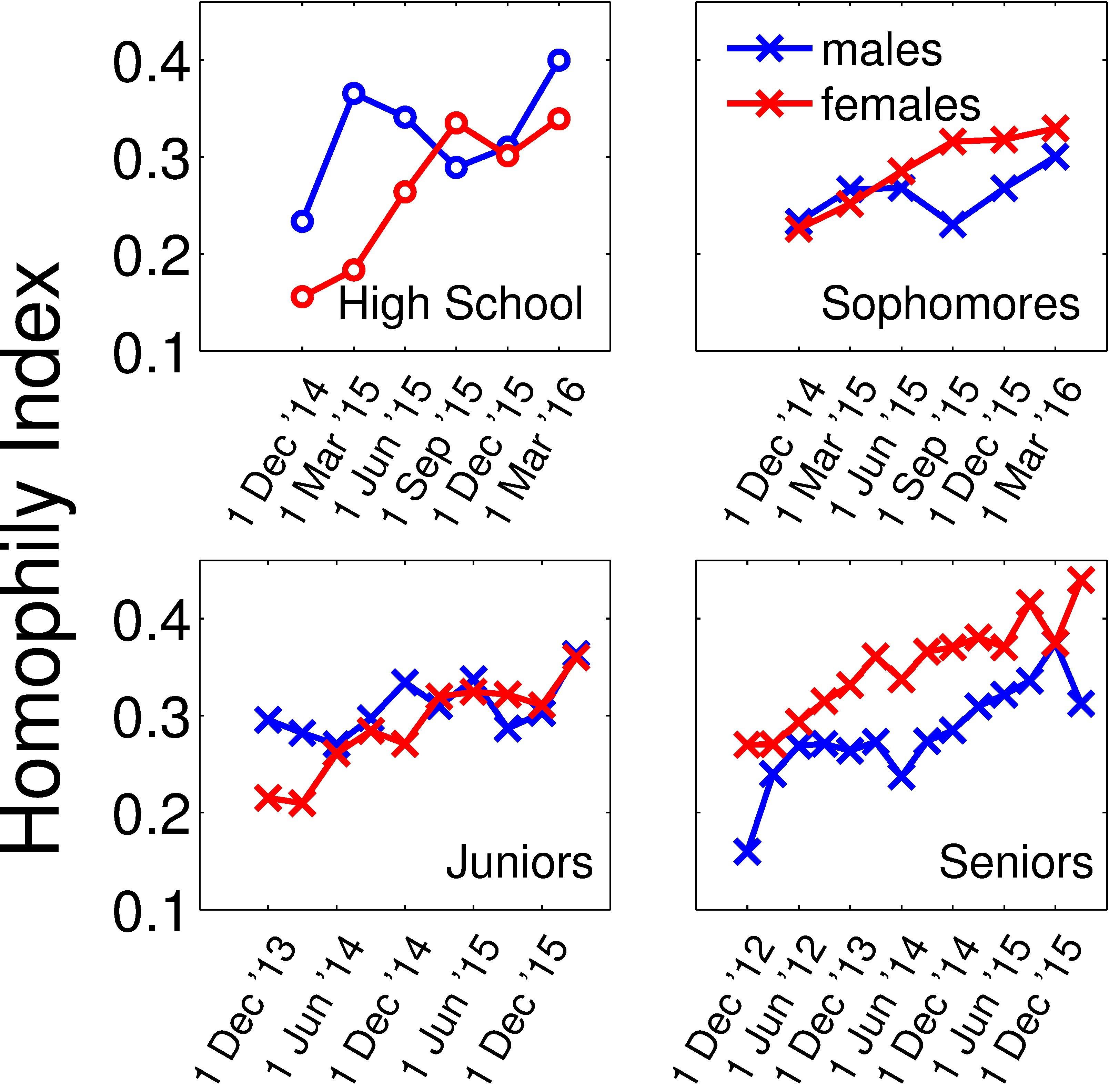

Supplement: S4 Fig — While both genders show about the same increase over time, it is larger for females in the sophomore and senior groups, and larger for males for the high school students and juniors. (TIF) [file pone.0183473.s005.tif]

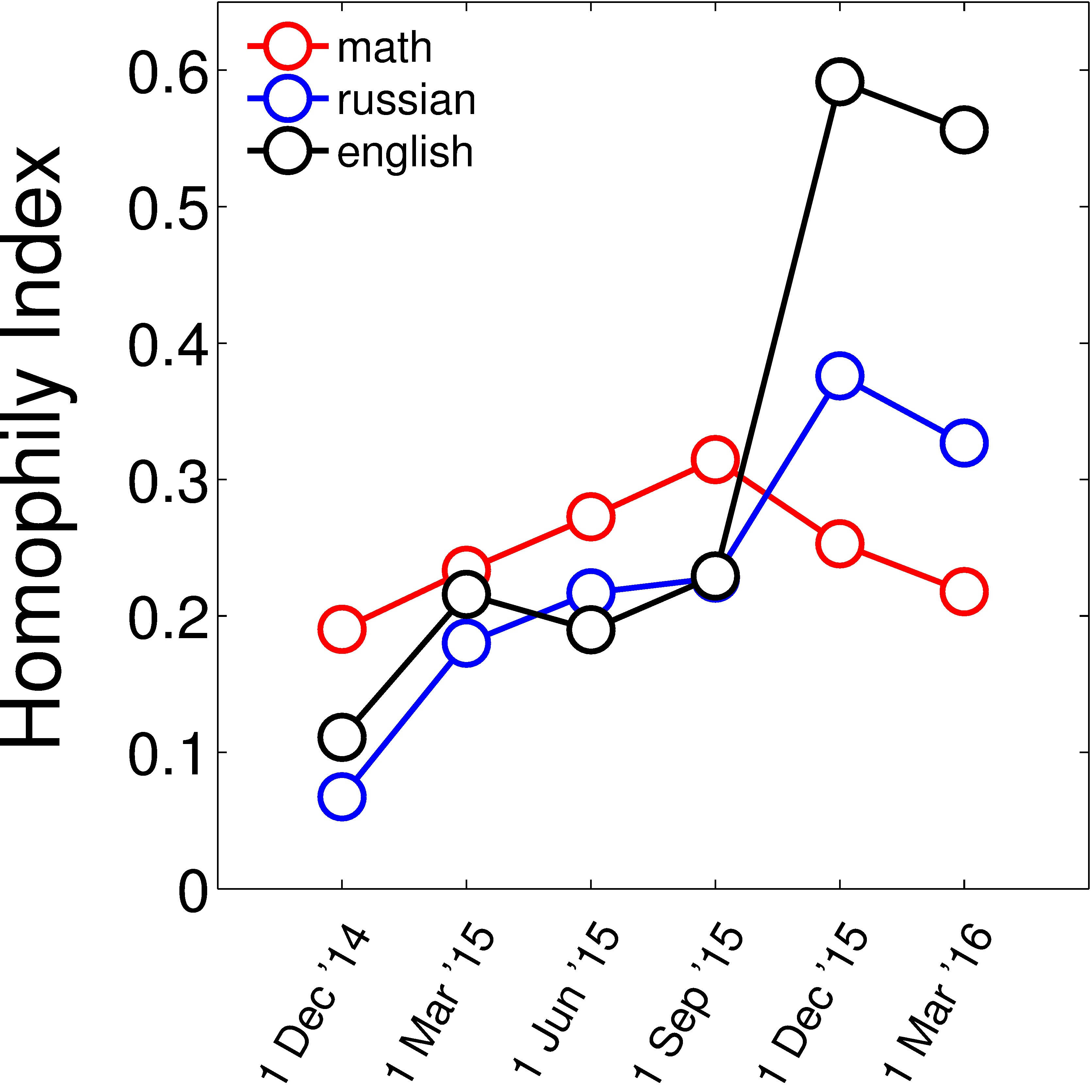

Supplement: S5 Fig — Since there are only 4 possible values of grades (scores) possible for the individual subjects, we expect to observe less stable results than for the GPA. However, the general pattern of homophily increase over time holds, for mathematics it is not much pronounced. (TIF) [file pone.0183473.s006.tif]

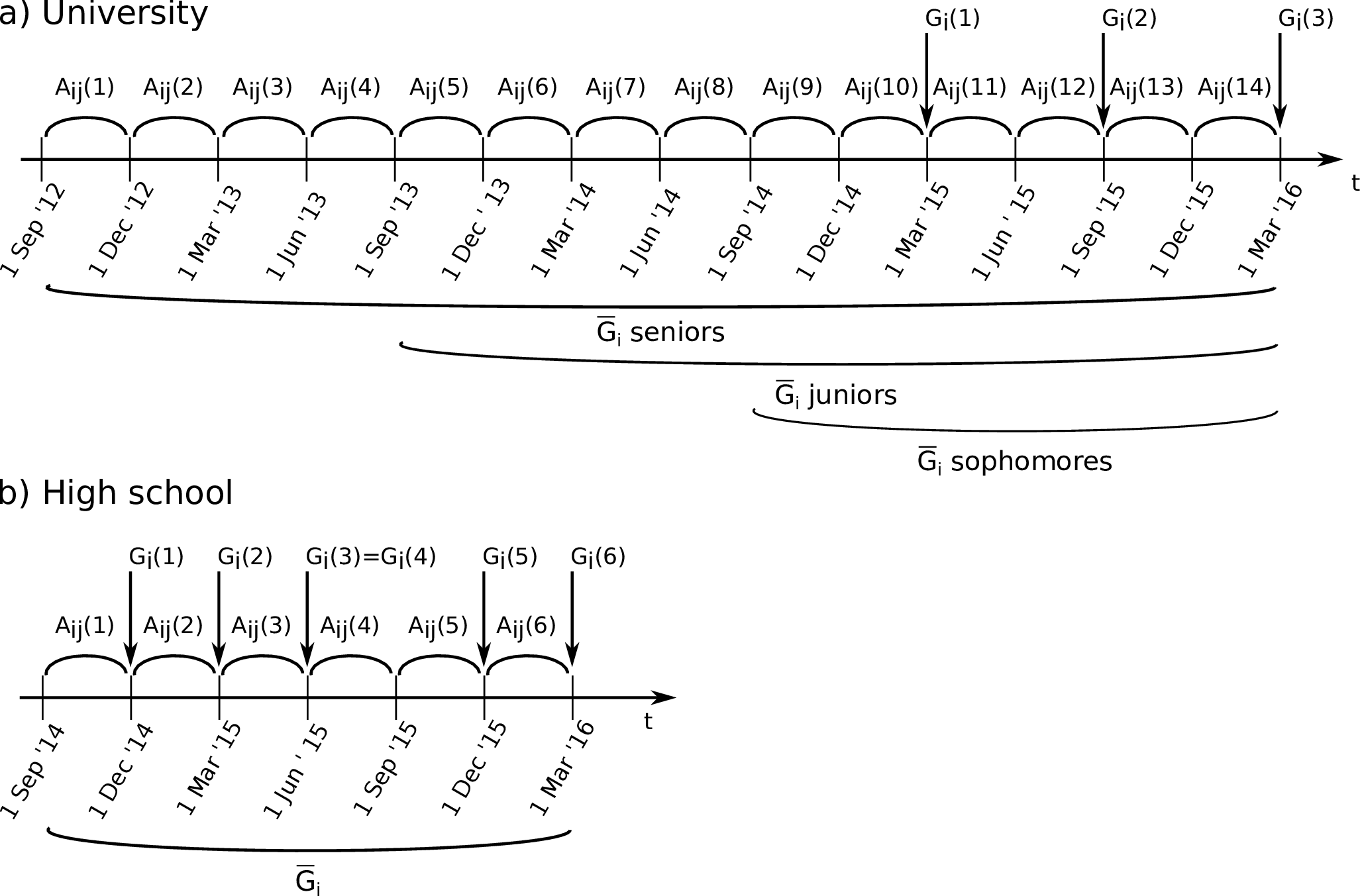

Supplement: S6 Fig — Network data is in the form of adjacency matrices Aij(t), where Aij(t) = 1 means that student i gave at least one “like” to student j from time t − 1 to time t. The time period from t − 1 to t is equal to 3 months. (a) For the university students (seniors, juniors, sophomores) the aggregated average GPA, G¯iU, from the beginning of their studies on the 1st of September (2012/2013/2014) until the 1st of March, 2016 is collected. This period is equal to 3.5 years for seniors, 2.5 years for juniors and 1.5 years for sophomores respectively. The temporal GPA data, GiU(t), was also collected for the last 3 semesters for all 3 cohorts (arrows). (b) For the high school students the temporal GPA data, GiHS(t), is collected at the end of each trimester for the last 5 trimesters (arrows). As students do not study in summer, we assume the same performance at that period as at the last available time point i.e. spring, GiHS(3)=GiHS(4). G¯iHS is computed as the average over the 5 trimesters. (TIF) [file pone.0183473.s007.tif]
