# Supplementary material for: Formation of homophily in academic performance: Students change their friends rather than performance
Source: PLoS One. 2017 Aug 30;12(8):e0183473. doi: 10.1371/journal.pone.0183473 (PMC5576666; doi:10.1371/journal.pone.0183473)
Supplement: S1 Table — 〈.〉i means average over all students in the group. Mean values and standard deviations (in brackets) are presented. Females have better grades than males on average. (PDF) [file pone.0183473.s008.pdf]

Table S1. Descriptive statistics of students' GPA scores across the whole period of their studies

	All students	Females	Males
$\langle G_i^{\mathrm{HS}} \rangle_i$	3.85 (0.53)	3.99 (0.53)	3.73 (0.50)
$\langle \bar{G}_i^{\mathrm{U},1} \rangle_i$	7.32 (1.02)	7.45 (0.97)	7.20 (1.06)
$\langle \bar{G}_i^{\mathrm{U},2} \rangle_i$	7.01 (1.11)	7.25 (1.05)	6.71 (1.11)
$\langle \bar{G}_i^{\mathrm{U},3} \rangle_i$	7.40 (1.26)	7.69 (1.15)	7.09 (1.29)
$\langle \bar{G}_i^{\mathrm{U},4} \rangle_i$	7.35 (1.21)	7.68 (1.10)	6.98 (1.23)

$\langle . \rangle_i$ means average over all students in the group. Mean values and standard deviations (in brackets) are presented. Females have better grades than males on average.
