# Supplementary material for: Formation of homophily in academic performance: Students change their friends rather than performance
Source: PLoS One. 2017 Aug 30;12(8):e0183473. doi: 10.1371/journal.pone.0183473 (PMC5576666; doi:10.1371/journal.pone.0183473)
Supplement: S2 Table — The GPA at the current time point is almost fully explained by the GPA at the previous time point. The influence of gender is also significant, males have lower grades also after controlling for their previous GPA. The average GPA of friends at the previous time point is not significant. (PDF) [file pone.0183473.s009.pdf]

Table S2. Coefficients from the regression model

	Parameter	Estimate	Std.Err	p-value
High School	α_1^{*****}	0.905	0.013	$< 10^{-15}$
	α_2	-0.003	0.006	0.56
	γ^{***}	-0.030	0.009	$< 10^{-3}$
	β (5th year)	0.020	0.015	0.18
	6th year	-0.022	0.016	0.17
	7th year	0.012	0.014	0.37
	8th year	-0.025	0.013	0.06
	9th year	-0.026	0.013	0.05
	10th year	-0.030	0.023	0.19
	c^{*****}	0.401	0.061	$< 10^{-10}$
University	α_1^{*****}	0.879	0.009	$< 10^{-15}$
	α_2	0.005	0.012	0.67
	γ^{***}	-0.103	0.018	$< 10^{-7}$
	β (3rd year)	0.144	0.025	$< 10^{-7}$
	4th year	0.022	0.027	0.65
	c^{*****}	0.769	0.100	$< 10^{-13}$

The GPA at the current time point is almost fully explained by the GPA at the previous time point. The influence of gender is also significant, males have lower grades also after controlling for their previous GPA. The average GPA of friends at the previous time point is not significant.
