# Supplementary material for: Formation of homophily in academic performance: Students change their friends rather than performance
Source: PLoS One. 2017 Aug 30;12(8):e0183473. doi: 10.1371/journal.pone.0183473 (PMC5576666; doi:10.1371/journal.pone.0183473)
Supplement: S3 Table — The GPA distance for new friends is consistently and significantly smaller (tested with two-sample Students’ test) than the GPA distance for discontinued friends in the observed data. Comparable results are obtained with the model. (PDF) [file pone.0183473.s010.pdf]

Table S3. Re-organization of the students' network over time

		D^{disc}	D^{new}	p-value
High School	Data	0.56	0.53	0.02
Model	$\theta = 0.55$	0.57	0.52	0.004
Sophomores	Data	1.17	1.08	$< 10^{-8}$
Model	$\theta = 0.60$	1.20	1.10	$< 10^{-7}$
Juniors	Data	1.22	1.14	$< 10^{-5}$
Model	$\theta = 0.61$	1.25	1.17	$< 10^{-5}$
Seniors	Data	1.22	1.11	$< 10^{-8}$
Model	$\theta = 0.58$	1.25	1.13	$< 10^{-7}$

The GPA distance for new friends is consistently and significantly smaller (tested with two-sample Students' test) than the GPA distance for discontinued friends in the observed data. Comparable results are obtained with the model.
